# 

**Published:** 1919-11-15

**Authors:** 


### Gifts and Bequests.

Amongst the Manchester charities that share in the residue of the estate of the late Mr. George Sampson are the Royal Infirmary, St. Mary's Hospitals, Lock Hospital, and Ancoats Hospital.

Under the will of the late Mr. Arthur Jennings, of Warwick, £1,250 has been bequeathed to the General Hospital, Birmingham, and £1,000 to the Birmingham and Midland Free Home for Children.

Mr. W. J. Rees, a former Mayor of Swansea, has left £200 to the Swansea Hospital and £50 each to the Deaf and Dumb Institution, the Institution for the Blind, and the Orphan Home in that city.

Sir William Vernon, Bart., of Chester, has left £500 to the North Staffordshire Infirmary, Hartshill.

Mr. Robert Gladstone, of Liverpool, ex-chairman of the Mersey Dock and Harbour Board, left £100 each (free of legacy duty) to the Liverpool Royal Infirmary, the David Lewis Northern Hospital, and the Royal Southern Hospital.

Under the will of Mr. R. H. Dunn, of Glasgow, charities in that city that benefit are the Glasgow Royal Infirmary, the Western Infirmary, the Victoria Infirmary, the Royal Hospital for Sick Children, £1,000 each. There are a number of other bequests of equal and smaller amounts, including £250 to the St. Andrew's Ambulance Association.

### MATRONS' AND SISTERS' APPOINTMENTS.

Baby Hospital, Glastonbury.—Miss R. C. Soutar has been appointed sister-in-charge. She was trained at the Royal Hospital, Aberdeen, and Gray's Hospital, Elgin, and has since been sister, Craiglockhart Hospital, Edinburgh, and matron Cottage Hospital, Kelso.

High Teams Institution, Gateshead-on-Tyne.—Miss Hall has been appointed night sister. She was trained at Gateshead Union Infirmary, and has since been staff nurse at Tooting Military Hospital.

Ilford Isolation Hospital, Chadwell Heath, Essex.—Miss L. M. Robinson has been appointed night sister. She was trained at Plaistow Fever Hospital, London, and at the County Hospital, Lincoln; she has since been attached to Q.A.I.M.N.S.R. at the Fargo Military Hospital, Salisbury Plain.

The Workhouse, Gateshead.—Miss Minnie Hall has been appointed night superintendent nurse. She was trained at the Gateshead Union Infirmary, where she became staff nurse, and has since been on service in war hospitals.

St. Martin's Hospital for Paralysed Soldiers, Cheltenham.—Miss M. Wright has been appointed night sister. She was trained at Queen's Hospital, Birmingham, and has been sister, Auxiliary Hospital, Downham Market, Norfolk.

### The College of Nursing (Limited by Guarantee).

EDINBURGH CENTRE.

Annual Meeting—Alteration of Place.

Wednesday, November 19, 3.30 p.m.—The annual meeting will be held in the Goold Hall, 5 St. Andrew Square, instead of the Edinburgh Café, as previously announced. Miss Gill, R.R.C., will preside. Tea will be provided. Members are again reminded that subscriptions for the ensuing year are now due.

